# Mixed methods evaluation of a primary eye care training programme for primary health workers in Morogoro Tanzania

**DOI:** 10.1186/s12912-016-0163-5

**Published:** 2016-07-07

**Authors:** Milka Madaha Mafwiri, Emma Jolley, Joanna Hunter, Clare Elizabeth Gilbert, Elena Schmidt

**Affiliations:** Department of Ophthalmology, Muhimbili University of Health and Allied Sciences, P.O. Box 65405, Dar-es-Salaam, Tanzania; Sightsavers, London, UK; University of Leeds, Leeds, UK; International Centre for Eye Health, Department of Clinical Research, London School of Hygiene & Tropical Medicine, London, UK

**Keywords:** Primary eye care, Primary health workers, Training

## Abstract

**Background:**

There are 285 million people with visual impairment (VI) worldwide including 39 million who are blind; 15 % of those with VI live in Africa, and around 80 % of VI is preventable or treatable with the right equipment, information and skills. The scarcity of human resources for eye health, particularly in Sub-Saharan Africa, is a key challenge towards achieving this goal. Therefore training primary health workers (PHW) in providing eye-care services has been seen by some authors as a way to improve access to eye-care services in remote communities. However, the package of interventions which could be effectively delivered for eye-care at the primary-care level or the set of skills and competencies that PHWs need has not yet been delineated. The aim of the study was to evaluate the effectiveness of a four day training programme of PHWs in primary eye-care conducted in Morogoro, Tanzania in 2010/2011.

**Methods:**

A mixed methods study using pre- and immediate post-training knowledge assessment of 60 trainees, and in-depth face to face interviews with 20 PHWs and 8 service managers 2 to 3 years after the training.

**Results:**

Pre-and immediate post-training assessments indicated improvement in health worker knowledge about eye-care in the short term. Qualitative investigations 2 to 3 years after the training showed that although staff could make the correct management decisions when presented with eye-health problems, they often could not make a correct diagnosis. PHWs and managers reported satisfaction with the content of the training but some of the less well qualified staff found it overwhelming. Theoretical teaching was appreciated by most participants but almost all suggested increasing the time spent on acquiring skills. The training manual was accepted by many and some improvements were recommended. All interviewed PHWs were keen to improve their skills and knowledge. Acquired skills and knowledge were used for identification, referral of patients and for eye-health promotion.

**Conclusion:**

The training program in Morogoro was considered by PHWs as broadly successful and satisfying in terms of content, methods and duration of training. However, any future programme needs to be considered within the context of strengthening wider health systems.

## Background

Globally, visual impairment affects 285 million people, 39 million of whom are blind [1]. Middle and low income countries account for 90 % of the global burden [2] and 15 % of the burden is in Africa [3]. With the right interventions up to 80 % of visual impairment is either preventable or treatable [3].

The 2014–2019 Global Action Plan, which was approved by the World Health Assembly in May 2013, aims to reduce avoidable visual impairment by 25 % by 2019 by ‘improving access to comprehensive eye care services that are integrated into health systems’ [1]. However, the limited number of eye care facilities and the scarcity of human resources for eye health, particularly in Sub-Saharan Africa (SSA)is a major impediment [4, 5].

Primary eye care(PEC) has been promoted by some authors as a strategy to address the burden of visual impairment in remote parts of Africa [6]. PEC has been defined as an integrated, participatory and inclusive approach to the eye health component of primary health care (PHC), consisting of promotive, preventive, curative and rehabilitative services [7]. It has been argued that by providing a package of basic services at health facilities close to where the majority of the population live, primary health workers (PHWs) can identify eye conditions early and either treat or refer patients in a timely manner and hence improve the eye health of communities. It has also been suggested that treating simple diseases in primary health facilities (PHFs) will free up the time of specialist staff at referral centres, enabling them to concentrate on more complex cases. Benefits to patients would include less time and money for travel. There is therefore an expectation that PEC would play a significant role in reducing visual impairment as a frontline activity, providing care and identifying diseases before they become serious medical issues [7]. Another potential benefit is that locally available services for eye care may reduce the use of harmful traditional eye remedies and inappropriate treatment obtained from untrained informal drug sellers.

However, the concept of PEC is not well developed and there is no consensus concerning what the package of services should consist of, who should deliver the package and how it should be structured within the broader health system. One way of delivering PEC has been training PHWs in eye health and integration of simple eye care interventions into the existing PHFs. Although there have been several recent initiatives in PEC, evidence of effectiveness remains weak [8]. Some of the key challenges identified include(a) lack of clarity and unrealistic expectations about what services can be provided at the primary level and what can be taught to the different cadres working at those levels; (b) lack of functional referral pathways to adequately equipped specialist services (c) lack of agreement on competencies and capabilities appropriate for different cadres of staff, and hence lack of standardised training curricula, methods of teaching and assessments and d) inadequate enabling environments, including supportive supervision, continuing professional development and mechanisms for quality improvement [9, 10].

The lack of consensus on PEC among the eye health community has undoubtedly undermined the ability to develop, test and roll-out appropriate and effective training courses to PHWs providing frontline eye services. Thus, a recent study in Kenya, Malawi and Tanzania found that although almost all PHWs provided some form of eye care, only a third to two thirds had ever received any training in eye health. Only 2 % to 8 % could measure visual acuity, a basic skill in eye care [11]. A study among PHWs delivering basic eye care in Tanzania found low levels of knowledge and ophthalmic skills among trainees, with a mean score of 6.12 out of a possible 12 with little association between receiving training and skills score [12]. However, another study in Tanzania where PHW delivered primary healthcare for children showed that although PHW’s initial knowledge of eye health was low, a short training course was effective in improving knowledge and changed practices in the short term [13].

A limited number of studies assessing the effectiveness of PEC training and the lack of homogeneity in training approaches in existing studies mean that no firm conclusions can be drawn about what works. In addition, few studies have explored the views of the PHWs trained in eye health and the impact the training had on their knowledge, skills and day-to-day practices.

The aim of this study was to evaluate the training of PHWs in eye health in Morogoro region, Tanzania. More specifically the study assessed (a) how PHWs understood the concept of PEC, (b) how they perceived the training received in terms of content, materials and teaching methodologies; (c) whether PHWs’ knowledge and skills changed after the training and (c) the outcome of training in terms of clinical management or health education practices.

### Study setting

Morogoro is one of 30 regions in Tanzania with a population of around 2.2 million people [14]. Although information on population level ocular morbidities in the country is sparse, eye diseases are among the top 10 commonest reported problems in facilities in Morogoro region [15]. Although no population-based surveys have been conducted in Morogoro, a rapid assessments of avoidable blindness survey (RAABs) in Kilimanjaro region and Zanzibar in 2007 indicate that the prevalence of blindness (presenting visual acuity of less than 3/60 in the better eye) among people over 50 may vary from 2.4 % to 3.7 %, and low vision may be around 6.4 %. Cataract is responsible for between a half and two thirds of blindness in the over 50s [16, 17].

Like other regions in Tanzania, health care in Morogoro is provided at four levels with referral pathways from dispensary through to tertiary centres. Dispensaries are the first contact of patients in the health system. There are about 261 dispensaries in Morogoro each covering approximately 10,000 population. Dispensaries refer patients to health centres (35 in the region) which cover a population of about 68,000 people. Health centres refer patients to 5 district hospitals. Patients from the district hospitals are referred to one region hospital. The tertiary eye referral centres for Morogoro are Muhimbili National Hospital and The Comprehensive Community Based Rehabilitation in Tanzania hospital both of which are located in Dar-es-Salaam about 200 kilometres from Morogoro. Eye-care services are provided at the district, regional and tertiary referral hospitals. Primary health workers provide services at the dispensaries and health centres [18].

A recent assessment of human resources for eye health in Africa identified that Tanzania is well below the recommended levels for ophthalmologists and eye surgeons (1.6 per million population versus the target of 4 per million), mid-level ophthalmic staff (3.8 per million versus the target of 10 per million) and optometrists/refractions (7.3 per million versus the target of 20 per million) [19]. The national cataract surgical rate (CSR defined as the number of surgeries per million population per year) is only a quarter of the recommended level (500 versus the target of 2000 per million) and the average number of cataract surgeries per surgeon is two thirds of the recommended target (340 versus 500 a year). Projections accounting for population growth, as the average number of surgeries per surgeon and the national CSR are all set to decrease. Additionally two thirds of all country ophthalmologists work in the capital Dar-es-Salaam, where only 7 % of the population live, leaving the rest of the population with limited access to care, if any at all. If eye care services do not expand commensurately with population growth and ageing the magnitude of vision impairment in Tanzania is likely to increase significantly over the coming years. In this context, the imperative arises to identify effective strategies to address the growing challenge of visual impairment in the country.

### Primary eye care training in Morogoro

In 2010/2011 one hundred sixteen PHWs received training in PEC in two districts in Morogoro region (Morogoro Rural and Mvomero). The main objective of the training was to improve access to basic eye care by early identification, treatment and referral of eye diseases.

Trainees were selected from all government PHF (health centres and dispensaries) in the two districts. One member of staff, usually clinical officers, was selected from every PHF in the two districts. In facilities where COs were not available, nurses (registered and enrolled), assistant clinical officers or medical attendants attended the training.

The training, which lasted for four days, was delivered in five workshops with around 20–25 participants in each workshop. Training was led by an ophthalmologist from the national tertiary hospital, assisted by an Assistant Medical Officer in Ophthalmology (AMO-O) and cataract surgeon from the regional hospital in Morogoro. The course focused on raising awareness of eye conditions, teaching basic diagnostic and treatment skills, and which patients should be referred to specialist eye care facilities. Participants were also taught health promotion messages which focussed on the importance of timely health seeking behaviour and the dangers of harmful traditional remedies. Practical training included taking a medical history, measurement of visual acuity, examining the eye and administering eye drops. After the training each participant received a training manual, a visual acuity chart and a torch.

The training manual was developed by the Ministry of Health and Social Welfare (MoHSW) with inputs from other eye health stakeholders and external consultants. It was written in English and illustrated with a number of photographs. Each section had an introductory overview, a description of how to address each eye condition in practice, trainers’ notes and a summary box of ‘points to remember’. The sections were (1) basic anatomy of the eye; (2) examination of the eye; (3) measuring visual acuity; (4) common abnormalities of the eye; (5) causes of acute red eye; (6) other causes of red eyes; (7) blindness; (8) refractive errors; (9) other eye problems, and (10) a summary on prevention of blindness.

Teaching methods included interactive lectures and group work delivered in Swahili. Practical skills training were delivered in the eye unit in Morogoro regional hospital. Due to the different educational background of trainees, the training approach was tailored to ensure each participant understood the course to their best ability. Participants in the first workshop also designed record keeping and referral forms which were included as appendices in the manual for subsequent trainees.

## Methods

The study used mixed methods. Quantitative data were obtained from a comparison of the PHWs’ pre- and post-training assessments which were undertaken immediately before and after the training in 2010/2011. This assessment focused on knowledge of eye diseases. In 2013, to understand whether and how that knowledge changed over the course of training, a qualitative study was undertaken which entailed interviews with 20 PHWs who had been trained and eight key informants. During interviews with PHWs diagnostic and disease management skills were assessed using descriptions of five clinical vignettes.

### Pre- and post- training knowledge test

At the time of the study only 60 matched pre- and post- assessments of the 116 originally completed questionnaires (51.7 %) could be obtained. The observed differences in knowledge scores were analysed using the paired *t*-test [20].

### Qualitative interviews

A total of twenty 20 trained PHWs (10 males and 10 females) were purposefully sampled for the study. Ten individuals of different cadres (i.e. clinical officers or other carders), facility type (i.e. dispensary or health centre) and distance from the district and regional hospitals were purposefully selected for interview in each district. Eight key informants responsible for the training and coordination of eye services at the district, regional and national level were purposefully sampled. Three informants had attended the training and had general medical backgrounds and five had eye health specialist expertise (three of them were trainers).

In-depth interviews with PHWs were conducted in their place of work by two investigators with expertise in eye care using a semi-structured questionnaire, which asked about participants’ qualifications and skills, their experiences of training, their role in providing PEC, the types of eye health problems they saw at their facility, and any changes made in their practice after training. Five clinical vignettes describing common eye conditions were presented to test PHWs’ diagnostic and disease management skills. The vignettes presented the following common eye conditions: 1) sticky red eyes in a 10 year old child; 2) painful loss of vision in one eye in an elderly lady; 3) a white spot in a baby’s eye; 4) painless diminishing near vision in a 40 year old; and 5) painless gradual loss of vision in a 70 year old. All cases would require examination, information and referral, except the first, which could be treated at the facility with antibiotic eye drops. We recorded whether PHW’s understanding of the problem was correct, and whether they ultimately made the right management decision.

Key informants were asked about their views on PEC, their perception of the workshop and training materials, and whether they observed any changes in PHWs’ practice in terms of referrals or management of cases.

Interviews lasted an average of 40 min (ranged 15–64 min). Interviews were audio recorded and transcribed verbatim. Data were analysed thematically using NVivo 10 Software [21]. All identifiable information (names or places) was removed or recoded as pseudonyms to protect participants’ identities.

Ethical approval was obtained from the National Institute of Medical Research, Tanzania (Ref NIMR/HQ/R.8a/Vol.IX/1531). Participants were provided with an information sheet in English and Swahili and provided signed consent.

## Results

### Before and after training knowledge test

Among the 60 participants for whom we could obtain both pre- and post-training assessments, 56 (93 %) scored higher on the post-training assessment with a mean increase in knowledge scores of 17.5 % (median 17 %)(paired *t*-test statistic 11.6 with 59 degrees of freedom, *p* < 0.001).

### Characteristics of participants interviewed

An equal number of male and female PHWs were interviewed. There were more COs than other cadres, reflecting the make-up of the original workshops. Primary Health Workers had been in post for an average of 5 years. The majority (18, 90 %) worked in dispensaries and two worked in health centres, reflecting the ratio of these types of facilities in the region (Table 1). Among key informants, half were female, and the average length in their current post was 2 years (Table 2).

**Table 1:** Demographic and proffessional characteristics of the study population

Characteristic		Number (*n* = 20) (%)
Gender	Male	10 (50)
	Female	10 (50)
Working station	Morogoro Rural	10 (50)
	Mvomero	10 (50)
Professional qualification	Nurse	5 (25)
	Clinical Officer	15 (75)
Mean time in current position		5 years
Working facility type	Dispensary	18 (90)
	Health Centre	2 (10)
Has access to:	Visual acuity chart	20 (100)
	Torch	13 (65)
	Training manual	16 (80)
	Tetracycline	8 (40)

**Table 2:** Characteristics of key informant participants

Characteristic		Number (*n* = 8) (%)
Level of informant working station	District	4 (50)
	Regional	2 (25)
	National	2 (25)
Eye health specialist		5 (63)
Gender	Male	4 (50)
	Female	4 (50)
Mean time in current position		2 years*

*Information only for 5 participants

### Clinical vignettes

Around two thirds of the participants undertaking clinical vignettes test gave correct diagnoses of the condition presented and four in five identified the correct course of action. Each participant made an average of 3.15 correct diagnoses and recommended 4.05 correct actions (Table 3). Overall, participants scored higher on recommended actions than on the correct diagnosis. The vignettes with the least correct answers were those involving elderly patients (2 and 5), where no more than half of respondents correctly diagnosed the condition. The vignette with the most correct responses was that of a child with conjunctivitis.

**Table 3:** Responses to clinical eye health vignettes presented to PHWs

Vignettes:	Correct diagnosis	Correct course of action
1) A mother brings her 10 year old daughter saying that she has had red eyes for 2 days and her eyelids are stuck together in the morning.	16/20 (80 %)	18/20 (90 %)
2) An old lady attends the clinic complaining of a painful eye, and she says the vision has almost gone in that eye. When you look at her eyes, one eye is very red. There is no discharge.	9/20 (45 %)	16/20 (80 %)
3) A mother comes to you saying that she has seen a white spot in her 2 year old child’s eye.	14/20 (70 %)	17/20 (85 %)
4) A teacher attends saying that he is having difficulty marking school books in the evening. When you look at his eyes they look normal.	15/20 (75 %)	15/20 (75 %)
5) An elderly man attends saying that his vision is getting dimmer in both eyes, but there is no pain.	10/20 (50 %)	16/20 (80 %)

### The role of primary health care workers in eye care

Primary Health Workers described their role as the first step in providing eye-care to distinguish serious cases and those in need of specialised care, from less complicated eye problems that they could manage at the primary facility. Many considered health education an important part of their work. They reported delivering eye- health specific education talks and integrating eye health information into general health messages.

*“One of our lessons is health education, to educate … patients on how to protect themselves … and the importance of going to the dispensary.”(Male Clinical Officer)*

One of the key activities described by PHWs was prophylaxis against ophthalmia neonatorum. However, the practice reported varied considerably between facilities, with some PHWs reporting that they cleaned all babies’ eyes and instilled tetracycline ointment soon after delivery, others did not do this at all, or only cleaned the eyes of babies where they observed a discharge or an unusual appearance.

As most respondents were the only staff trained in eye care at their facility they attended the majority of eye cases. All identified a need to train more staff to cover staff sicknesses and staff turns over:

*“Sometimes you can find I am not here in the dispensary, maybe I went into town for other training or meetings. I hear sometimes my colleagues complaining that they don't know anything about eyes, even though I gave them the feedback but they still complain about it.” (Male Clinical Officer)*

### Perception of training

The training was well received by both the trainers and the trainees. The main factor contributing to participants’ satisfaction was gaining new knowledge and developing new professional skills which were particularly common among the trainees with no previous experience in eye health. Most said that their knowledge of eye conditions was limited and none reported receiving any refresher courses in eye health since their initial pre-service training.

*“Because from the beginning I didn’t understand about eye problems, so I learnt. It was good, I enjoyed.” (Key informant, trainee)*

*“After the training they [participants] were very happy, because they knew, especially on children, they knew how to [work] on children. During their basic professional training, children were not included at all… they got little information for children. After this, they were able to know problems, blindness, when to refer, when not to refer.” (Key informant, trainer)*

Some participants thought that the training focused more on raising awareness of eye diseases than on disease management skills. One of the key challenges identified was when mixed cadres were trained together trainers concentrated more on those who found the training more difficult due to their lower educational level. Although most trainees agreed that different cadres working in PHFs required eye health training, some, especially COs, expressed disappointment in the relatively low level of information provided. Many would have liked the course to go in to greater detail about different eye diseases and treatments. Some wished to undertake a longer course in eye care, which included basic surgical procedures such as lid surgery for trachomatous trichiasis:

*“I think because the training was not for…it was preliminary. But because the training was just a few days…” (Male Clinical Officer)*

Nearly all respondents felt that the training was too short and should be extended by at least an extra two, if not more, days and that additional time was needed to practice on eye patients rather than pictures.

*“The training was fine, but a very short time for practice, because … the theory was good, but practical is much better. Because theory you can forget sometimes but practical you can never forget.” (Male Clinical Officer)*

*“What I think is also that the time for the training wasn’t enough for them to absorb everything that we could do. We could do extended training for 3 more days, that could be used to take those participants to the nearby eye clinic and stay there for 3 days doing an eye screening, examining the patients, could have developed their skills better than what we did in that training.” (Key informant, trainer)*

Many interviewees emphasised the importance of refresher courses and continued professional development through in service training. It was felt that the courses should be tailored to the needs of those who required very basic knowledge and skills in eye health as well as those who wanted to improve their knowledge further.

*“If they conduct on-the-job training it would [be] good because it would help us to remember well. But if we just stay here then we will forget….there is a lot of work. So if we refresh then you are reminded and you get more experience.” (Female Nurse)*

*“Also, if we get retraining ………, at least after two to three years we get retraining and if it is important to get more, go deep so we can be most competent in dealing with the eye problems. That will be the most important things to me.” (Male Clinical Officer)*

The majority (16 out of 20) of participants still had the manual from the training course at the time of the evaluation. The manual was used as reference for themselves as well as for teaching colleagues. Although most participants were positive about the content of the manual, some argued it needed revisions and improvements. The manual required more in-depth information, better explanations, more appropriate photographs and disease management flowcharts. Some argued that a manual in Swahili would be more appropriate particularly for those not fluent in English. Others argued that the materials in Swahili required better translation.

*“The changes it needs because the translation of this one is just shallow, not more elaborated, just mentioned other diseases: red eyes just mentioned, not elaborated… even in Swahili.” (Male Clinical Officer)*

Some said that manuals for other diseases described relevant eye conditions (e.g. ophthalmia neonatorum in the Sexually Transmitted Infections manual) and advised that these could be used in future PEC training.

### Post-training changes

Most PHWs and key informants noted changes in management practices following the training. The key changes reported by PHWs included greater understanding of how to examine eyes and to test visual acuity:

*“…to look at the eyesight – long sighted, short sighted, how to close the eye and read the alphabet there on the wall… That’s the most [important] thing that was new to me. … That’s for me the most important.” (Male Clinical Officer)*

*“..Before that … I didn’t know even [how] to look at the cataract, [how] to use a torch to look at the eye. But then after, things go well.”(Female Clinical Officer)*

Many PHWs reported significant improvement in knowledge in relation to which eye diseases can and cannot be treated.

*“I didn't know that squint was curable but now I know, so now I can help a child when I see one with a squint … And lots of other problems, the drooping eyelid [ptosis], I didn't know there was treatment so it has changed a lot.” (Female Clinical Officer)*

PHWs reported that they better trained to correctly diagnose eye diseases and treat conditions which could be managed at the PHF level:

*“Before the training? Sometimes we misdiagnosed it. Because there is not enough knowledge to know the problem, sometimes you can provide the drugs which is not effective … But I sense after getting training, I get knowledge, so if a patient comes I know what to do.” (Female Clinical Officer)*

*Before I didn't understand……, before a patient might come having been hit here [refers to forehead just above the eye]. I knew this was trauma but I didn't know it involves the eye, now I know to look at the eye. But before I didn't know this stuff…I detect it very fast because I have had training.”(Female Nurse)*

A large number of PHWs argued that their knowledge of which conditions to refer had significantly improved and that their decisions were better informed. This was corroborated by the key informants, who noted an increased number of referrals to secondary facilities in the post-training period, although no routine data could be systematically collected to verify these claims:

*“… Previously we were treating and we were not looking at the needs for referral. We were doing just conjunctivitis and then refer. But now we are looking [at] everything, if this is cataract then refer, if any foreign body then, formally we were giving eye antibiotics, but after this training we were taught we are not allowed to perform anything on eye with a foreign body. Eye treatment, if anything, or any insect has entered the eye it could have traumatised the eye so it’s supposed to be referred to the eye clinic to make more examination and if need to be, to do surgery to remove foreign body.” (Male Clinical Officer)*

*“I see the referral forms from the villages, from the ones who get the trainings… At last they increased…there are many changes.” (Key informant)*

The increased knowledge and skills described by the PHWs had also led to increased confidence and satisfaction with the service they provided to their communities. Respondents described overcoming fear and feeling responsibility towards patients.

*“From the beginning I didn’t know anything. But from the training I get knowledge. From the beginning I have fear if a man comes with [an eye disease]. How can I understand? What should I do? But now I have confidence. If a man came I can treat.”(Female Clinical Officer)*

Following the training, many PHWs assumed positions of expertise within their PHFs and referred patients with eye diseases regardless of their qualifications. Many found it motivational and inspirational and wished to develop their knowledge further. Several PHWs developed their own eye information posters, some in English and others in Swahili, which led to a better recognition of eye care issues in their facility as a whole:

*“In eye problems I just feel like I am more responsible because I am the one who had the training on it. As I am a human being I feel like I am the one who understands more than my colleagues. Before no one knew it, but now I feel like I know it.” (Female Clinical Officer)*

However, despite the overall improvements in eye care management, both PHWs and key informants acknowledged that problems with the health system constrained opportunities to provide the best care possible. These included lack of equipment and supplies, being overworked, and a poorly functioning referral system.

*“Somehow I have changed but due to facilities, problems with facilities, sometimes there are no drugs, examining instruments.”(Male Clinical Officer)*

*“It’s half half….They’re doing a good job. They are doing what we told them, but they are facing challenges, which is why I am saying half half… Because they make diagnosis correctly but find that they don’t have the medicine for that patient in the health facilities. They may diagnose the patient correctly and decide to refer the patient, but in the end they find that the referral system is not working.” (Key informant)*

Some participants argued that the local Health Management Information Systems (HMIS) required adaptations as they were not suitable to record a variety of eye diseases which PHWs were now able to identify.

Observations of clinical practices in the facilities at the time of the evaluation showed that although all interviewed staff had access to a visual acuity chart, only two thirds had access to a torch for basic eye examinations. Most torches were purchased by the health care workers themselves. Only 8 out of 20 participants had access to tetracycline in their facility.

## Discussion

This qualitative study found that the PHWs and health care managers in Morogoro considered the PEC training to have been broadly successful. The training course fulfilled its aims in terms of increasing the awareness of PHWs about eye health. The data from the pre- and post- training assessment also indicates that the training was successful in increasing the knowledge of the PHWs about eye diseases, although this finding is significantly undermined by the fact that only half of the completed assessments were available at the time of this evaluation. In addition, the post-training assessment scores were measured immediately after the training and we are not able to say how sustainable this change in knowledge was.

The study shows that most trained PHWs broadly understood what PEC was and what role they could play in improving population eye health. The PHWs’ role was seen largely as raising awareness of eye problems, education of patients, diagnosing eye diseases, managing conditions that could be treated in PHFs and referring those requiring specialist care. Responses to the clinical vignettes showed that participants were more likely to appropriately refer patients than to correctly diagnose the condition. The evidence from the qualitative interviews also suggest that the four day training conducted in this setting was more effective in raising awareness of eye health than imparting skills in clinical management. This finding is consistent with evidence from earlier research in Tanzania and other settings in Africa, which showed that no more than two thirds of PHWs trained in PEC could identify cataract in pictures [12] and the PHWs’ ability and willingness to measure visual acuity, remove foreign bodies or accurately differentiate the causes of a red eye did not change after training [22]. Whether the knowledge and skills acquired during training will be translated into action in the long term will depend on overcoming the multiple barriers to this process [23].

It is difficult to say to what extent the outcomes of a particular training programme are determined by the content and quality of the programme itself and to what extent by the qualifications and preparedness of the trainees. We found evidence that both factors may play a role. Thus, although the content of the PEC programme evaluated here was well perceived and satisfied a number of PHWs, many felt the training time to be too short, particularly for practical learning of skills, such as measuring visual acuity, examining the eye, installation of eye drops, bandaging the eye and removal of foreign bodies. It has long been recognised that practical training is more effective than didactic classroom teaching alone [24]. Nearly all participants in this study expressed a wish for more varied practical examples and longer training on patients in a clinical setting.

The differences in prior training and experiences of trainees participating in this programme resulted in some PHWs being overwhelmed by the information provided, whereas others wished more detailed and advanced content. The training course was planned for PHWs who had professional qualifications as COs and nurses. However, the human resource crisis experienced by most Sub-Saharan African countries including Tanzania meant that training lower cadre participants was the only option, as they were the only staff available in some facilities. Given that the lack of appropriately qualified human resources is common in many settings in Africa, future PEC programmes should consider whether all PHWs are expected to deliver the same package of eye care interventions irrespective of their initial education level. Also, PHWs with different professional qualifications should be trained separately, as trainees with the very basic level of clinical knowledge and skills would require more tailored teaching and longer courses.

Primary Health Workers were generally satisfied with the training materials provided. However, some thought the manual was not comprehensive enough and lacked teaching tools such as simple diagnostic and management guidelines. Preparation of training materials is important for the success of any training program [25]. The manual used in this programme had been pre- tested with potential trainees in Dar-es-Salaam although trainees in this city may be better educated generally, and be able to access information more readily from other sources. More participatory approaches to the development of future training materials can help organisers improve the quality of the teaching resources in terms of the structure of manuals, use of language and appropriate selection of photographs. It is also advisable to review other materials and treatment protocols currently available in Tanzania, to avoid replication and ensure more standardized approach to disease management.

One important finding of this evaluation was increased clinical confidence and motivation among PHWs who undertook the course. The PHWs interviewed were enthusiastic about being able to deliver a better standard of care within their communities and many appeared keen to provide even more services. Another positive finding was a reported increase in the number of referrals to specialist services, although in the absence of any control group and without any data on referrals by the PHWs in the pre-training period, we cannot definitively attribute the observed change to the training.

Although both high levels of morale among health care workers and high demand for care among patients are essential elements of an effectively functioning health system, some potential effects of PEC expansion on the wider health system need to be considered [26]. First, some studies have showed that PHWs in SSA are already overburdened by other responsibilities including deliveries, immunisation and treating patients with HIV/AIDS [27]. Adding more duties within PHFs can add pressure on an already overstretched system and undermine the quality of other healthcare services. Second, given that the majority of patients with visual impairment require specialist services, expansion of eye care within PHFs may not necessarily result in reduced patient time or costs of care, particularly if PHWs are not adequately skilled or do not have appropriate medicines or equipment to treat even simple eye conditions. Third, the lack of specialist eye care services remains a serious problem in many parts of SSA. Increasing demand for services and referrals where services are not available raises ethical concerns.

## Conclusions

In conclusion this study suggests that training PHWs in eye care can have a positive effect on raising the profile of eye health services within health systems, increasing PHWs’ knowledge, confidence and motivation and increasing the number of referrals to specialist care. However, PEC training alone is unlikely to be a panacea for the limited availability and poor quality of services. Training PHWs cannot be effective without strengthening other elements of wider health systems, including availability of human resources, procurement of equipment and supplies, adequate HMIS and referral tracking. In addition, as the evidence underpinning primary eye care interventions remains scarce, future work should incorporate rigorous evaluations of both PHWs’ competences and the impact of PEC integration on access and quality of services.

## Abbreviations

AMO-O, Assistant Medical Officer in Ophthalmology; CO, Clinical Officer; CSR, Cataract Surgical Rate; HMIS, Health Management Information System; MoHSW, Ministry of Health and Social Welfare; PEC, Primary Eye Care; PHC, Primary Health Care; PHF, Primary Health Facilities; PHW, Primary Health Worker; RAAB, Rapid Assessment of Avoidable Blindness; SSA, Sub-Saharan Africa; VI, Visual impairment.
